# Gut microbiome remodeling induced by microplastic exposure in humans

**DOI:** 10.1080/19490976.2026.2617696

**Published:** 2026-01-19

**Authors:** Xin-Yue Yang, Zhong-Wei Zhang, Guang-Deng Chen, Shu Yuan

**Affiliations:** a International Science and Technology Cooperation Base for Efficient Utilization of Nutrient Resources and Fertilizer Innovation, College of Resources, Sichuan Agricultural University, Chengdu, People's Republic of China

**Keywords:** Microplastics, gut microbiota, pathobionts, probiotics, human diseases

## Abstract

The impact of microplastics (MPs) on the diversity and composition of the gut microbiome has been extensively documented in animal studies, but evidence in humans remains limited. Recognizing the potential differences in MP effects between animal and human gut microbiomes, this review synthesizes current evidence concerning their impact on the human gut microbiota. Furthermore, the potential links between microplastic-induced dysbiosis and the pathogenesis of human diseases were analyzed. Cross-sectional studies have been conducted to explore microplastic exposures (such as in humans who consume hot foods served in disposable plastic tableware) and their associations with gut microbiome functionalities in infants, preschool children and adults. Exposure to MPs increased the abundance of Dethiosulfovibrionaceae, Enterobacteriaceae, Moraxellaceae, Actinomycetota, Pseudomonadota, and *Veillonella*. On the other hand, MPs decreased the abundances of Bacillota, Bacteroidota, Lactobacillales, Rikenellaceae, *Parabacteroides*, *Roseburia*, *Coprococcus*, *Turicibacter*, and *Eubacterium coprostanoligenes*. These changes were associated with a decrease in butyrate production and a decrease in short-chain fatty acid levels. However, for some other bacteria, both inductive (on Oscillospiraceae, *Adlercreutzia*, *Phascolarctobacterium,* and *Collinsella*) and repressive effects (on *Streptococcus*) have been documented. There are contradictory reports about MP-induced changes in Lachnospiraceae (including the *Dorea* genus), *Alistipes* and *Faecalibacterium*, which may be correlated with obesity, gastrointestinal dysfunction, some cancers, inflammatory bowel disease and Crohn’s disease. Potential reasons for these discrepancies are proposed. This review also examines putative mechanisms, with a focus on biofilm formation on selective surfaces, and discusses the inherent limitations of current MP exposure assessments in human gut microbiota studies.

## Microplastics change animal and human gut microbiome differently

Microplastics (MPs) are omnipresent organic pollutants that are increasingly recognized for their harmful effects on gastrointestinal health. MP-induced gut microbiota dysbiosis may aggravate inflammatory responses and intestinal barrier dysfunction. Through these reciprocally connected pathways, long-term MP exposure may be correlated with the onset and development of gastrointestinal diseases.^1^ Over the past decades, MPs have been found in multiple human organs (such as the kidney, liver and placenta) and blood, which implies their ability to traverse the body.^2-8^

Although the influence of MPs on the biodiversity and composition of the animal gut microbiome has been widely reported,^9-12^ few human studies exist. Compared with actual human exposure conditions, experimental animal models usually adopt MPs at higher contents and shorter exposure periods.^13^ Though this short-term, high-level exposure may facilitate rapid measurements of biotoxicity, it may not reflect long-term but low-level exposure in humans. Moreover, limitations in present MP detection facilities make it difficult to determine the real content of human exposure.^14^ Therefore, the data from acute exposure to animal models may overstate the chronically toxic effects of MPs on humans. In experiments with animal models, the sizes of MPs are usually less than 5 μm in a spherical shape. Nevertheless, MPs in human tissue samples are usually larger than 50 μm in fiber, fragment, and film shapes with rough surfaces, which may not traverse the intestinal barrier as easily as small and regular MPs.^15^ Previous studies with animal models focused on a single type of MP, e.g., polyethylene or polystyrene. However, realistic MPs in the human body are mixtures of MPs, such as polyvinyl chloride, polypropylene, and polyethylene terephthalate, which may generate complex toxic effects. Furthermore, environmental MP pollutants usually include opportunistic pathogens and other co-pollutants, such as antibiotics, pesticides and heavy metals, which may potentially increase their toxicity or alter their immunological effects.^16^ On the other hand, the gut microbiota composition and the intestinal microenvironment in animal models are largely different from those in humans, which may also influence the biological effects of ingested MPs.^1^

## Putative mechanisms of microplastics-induced gut microbiota dysbiosis

MPs can act as carriers for co-pollutants such as persistent organic pollutants (POPs), heavy metals, antibiotics, and pathogens. This “Trojan horse” effect introduces significant toxicological complexity. A critical unresolved issue is whether probiotics that bind to MPs could unintentionally promote the uptake of these sorbed hazardous substances or pathogenic microbes.^17^ Although existing evidence highlights the protective role of probiotics, further research is needed to determine if microbe-MP complexes alter the bioaccessibility of toxicants or influence gut absorption pathways.^18^^,^^19^ This potential for dual effects must be accounted for when designing probiotic therapies.

The intestinal barrier consists of the gut microbiota, mucosal layer, tight junctions, and intestinal epithelial cells.^20^ Exposure to MPs can compromise this barrier by inducing reactive oxygen species (ROS)-mediated apoptosis in epithelial cells, which elevates permeability in the duodenum, jejunum, and colon.^21^ Studies in mice indicate that MPs-induced dysbiosis of the gut flora can induce the expression of inflammatory factors (e.g., interleukin-1β, tumor necrosis factor-*α*, interleukin-6, toll-like receptor 4, proinflammatory transcription factor activator protein 1, and interferon regulatory factor 5), contributing to barrier injury.^9^^,^^22-24^ Furthermore, ROS overproduction from MPs exposure down-regulates tight junction proteins (ZO-1, occludin, claudin-1)^25-27^ while up-regulating ion transport genes such as Na-K-2Cl co-transporter 1 (Nkcc1), Na^+^/H^+^ exchanger 3 (Nhe3), solute carrier family 26 member 6 (Slc26a6) in the ileum and colon, thereby increasing intestinal permeability.^28^ MPs-triggered inflammation can also suppress mucin secretion, impairing the defensive function of the mucus layer. The resulting gut microbiota dysbiosis further exacerbates barrier dysfunction,^29^ creating a vicious cycle in which endotoxin leakage activates inflammatory pathways, perpetuating a state of inflammation, dysbiosis, and impaired barrier integrity.^1^

Research has shown that biofilms can develop on microplastic surfaces. According to Galloway et al.^30^, microplastics frequently interact with proteins and other biomolecules in biological fluids, forming a protein corona layer. This nutrient-rich layer enhances the interaction between microplastics and living cells, promoting bacterial attachment and biofilm formation.^31^ Studies indicate that biofilms can alter microplastic properties by modifying carbonyl indices and double-bond concentrations.^32^ The combined presence of microplastics and biofilms increases environmental hazards and poses serious health risks.

The development of biofilms on microplastics depends on multiple factors, including particle size, shape, polymer composition, ionic characteristics, and heavy metal concentrations.^33^^,^^34^ Additionally, biofilms on microplastics can accumulate contaminants such as heavy metals and antibiotics. There is also evidence that pathogenic microbes colonize microplastic surfaces through biofilm formation. For instance, *Vibrio* spp. Other microbial species, including Alpha-Pseudomonadota (Sphingomonadaceae, Rhodobacteraceae, Devosiaceae) and Gamma-Pseudomonadota (Pseudomonas, Alteromonadaceae), have been detected on microplastics.^35^ These findings suggest that microplastics may act as vectors, transporting harmful pathogens and potentially introducing them into food chains, thereby increasing the risk of foodborne disease outbreaks.^36^

According to one study, the surfaces of MPs undergo significant changes during digestion. Characterization using Raman spectroscopy and field emission scanning electron microscopy revealed morphological alterations after the gastric phase. Later, during the intestinal and colonic phases, organic matter adhered to the MPs and a microbial biofilm formed, accompanied by surface biodegradation.^37^ Biofilms function as protective multicellular colonies that invade human tissues, shielding their cells from environmental threats such as antibiotics and immune responses.^38^ Research indicates that as plastics break down in the environment and the gut, the resulting MPs develop surfaces that are conducive to biofilm colonization.^39-42^ These biofilms can act as “rafts,” increasing the bioavailability and transport of opportunistic pathogens such as *Vibrio* spp. and *Escherichia coli*.^43-45^ The microbial communities on microplastic biofilms are distinct from those on natural substrates such as leaves or stones and have been found to include human pathogens such as *Pseudomonas monteilii* and *Pseudomonas mendocina*.^46^ Similarly, MPs can serve as vehicles for other pathogens, including *Helicobacter pylori* and *Fusobacterium* spp. While *H. pylori*, a group 1 carcinogen, causes inflammation that disrupts cellular processes such as the cell cycle and antioxidant defense.^47^^,^^48^ The presence of such pathogens is linked to gut dysbiosis, which impairs the microbiome's roles in metabolism, immunity, and neuroendocrine function.^47^^,^^48^ This imbalance can lead to conditions such as irritable bowel syndrome, leaky gut syndrome, and neurological disorders, including Parkinson's disease and multiple sclerosis.^49^ The establishment of a biofilm itself is a morphological change driven by bacterial components such as adhesin proteins and efflux pumps, which define the colony's structure and physiology.^50^

The surface of MPs acts as a substrate for biofilm development, which can amplify the harmful potential of pathogens by making them more pathogenic, antibiotic resistant, and carcinogenic. Gene expression analysis reveals that MPs stimulate the transcription of microbial genes involved in invasion, quorum sensing, and the biodegradation of plastics themselves.^51^ Research into the gastrointestinal effects of MPs has documented microbiota dysbiosis, oxidative stress, and inflammatory responses in regions like the duodenum, jejunum, ileum, and colon.^52^^,^^53^ The role of MPs in gene transfer is underscored by findings that gram-positive bacteria (e.g., *Bacillus subtilis*) in biofilms are more proficient at acquiring extracellular antibiotic resistance genes than their planktonic counterparts.^54^ Consequently, plastic surfaces serve as a crucial niche and a hotspot for horizontal gene transfer among microbes.^55^^,^^56^

## Microplastics inducible bacteria

**Figure 1.**
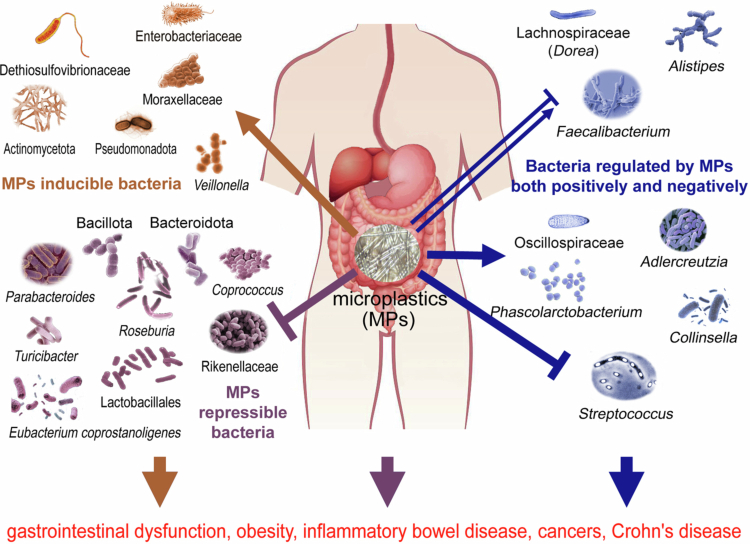
Microplastics induced gut microbiome remodeling in humans.

In experiments with Toddler mucosal Artificial Colon (Tm-ARCOL) bioreactors inoculated with fecal samples from the four infant donors, exposure to MPs induced gut microbial shifts, including an increase in abundance of Dethiosulfovibrionaceae, Enterobacteriaceae, and Moraxellaceae (Figure 1).^57^ This increase was associated with a decline in butyrate production and significant changes in volatile organic compound profiles.^57^ The Dethiosulfovibrionaceae family has been found to be correlated with colorectal cancer,^58^ while the Enterobacteriaceae family contains well-known enteric pathogens, such as *Campylobacter*, *Salmonella*, *Escherichia*, and *Shigella*, which has been found to be enhanced in irritable bowel syndrome (IBS)^59^ and other inflammatory diseases.^60^ *Moraxella catarrhalis* has defined its position as a key human mucosal bacterium, no longer being considered as only a commensal bacterium.^61^

In humans who consume hot foods served in disposable plastic tableware, their gut microbiota compositions presented an enhanced abundance of the phyla Pseudomonadota and Actinomycetota (Figure 1).^62^^,^^63^ Increases in Pseudomonadota and Actinomycetota have been observed in women with depression,^64^ and previous reports demonstrated that both of them include some steroid-degrading bacteria.^65^ Sexual hormones could be degraded by Pseudomonadota and Actinomycetota, which leads to lower levels of estrogens in women.^66^ Low estrogen levels have been suggested to be correlated with the onset of depression.^67^

In young adults who often consume take-out foods, high MPs exposure enhanced abundance of *Veillonella* (Figure 1).^68^ A previous study confirmed that overweight people have higher levels of *Veillonella*.^69^

## Microplastics repressible bacteria

MPs exposure was found to be associated with certain declines in some taxa, such as Lactobacillales, Rikenellaceae, *Parabacteroides*, and *Eubacterium coprostanoligenes* in preschool children (Figure 1).^70^ Those declines were correlated with decreases in butyrate production and short-chain fatty acid levels. A large number of Lactobacillales and Rikenellaceae bacteria show anti-inflammatory effects, and produce short-chain fatty acids, which present protective effects to the intestinal barrier.^70^ *Parabacteroides*, whose decrease was correlated with the onset of inflammation bowel disease (IBD).^71^^,^^72^ The *Eubacterium coprostanoligenes* group decreased after a high-fat-diet treatment in mice,^73^ implying its potential effect on lipolysis.

MPs exposure also reduced abundance of Bacillota, Bacteroidota and *Roseburia* (Figure 1).^62^ A previous report indicated a positive coordination between the level of Bacillota and Bacteroidota, which regulates the circadian clock and sleep quality of animals and humans via the production of butyrate, which decline is inextricably associated with depression and anxiety.^74^ Moreover, both Bacillota and Bacteroidota were reduced in people with generalized anxiety.^75^ Bacillota abundance was significantly lower in either Crohn's disease or ulcerative colitis patients; while Bacteroidota was only reduced in Crohn's disease patients.^76^ The genus *Roseburia* also produces butyrate in the colon, which could maintain energy homeostasis and suppress intestinal inflammation.^77^ On the contrary, declined levels of *Roseburia* have been observed in patients with IBD.^77^

*Coprococcus* and *Turicibacter* also decreased under MPs exposure (Figure 1).^68^ *Coprococcus* bacteria also play roles in maintaining the stability of microbiota networks and are important producers of butyric acids.^78^ *Turicibacter* bacteria, as another kind of intestinal probiotics, regulate lipid and sugar metabolism through producing polysaccharides.^79^

## Microplastics generate both inductive and repressive effects on some bacteria

However, for some other bacteria, both inductive (on Oscillospiraceae,^57^ *Adlercreutzia*,^68^ *Phascolarctobacterium,*^68^ and *Collinsella*^80^) and repressive effects (on *Streptococcus*^70^) were documented (Figure 1). The *Oscillospiraceae* population is positively related to Crohn's disease^80^ but negatively related to hepatitis B virus (HBV)-related hepatocellular carcinoma.^81^ *Adlercreutzia* has been suggested to be positively associated with Alzheimer’s disease.^82^ However, *Adlercreutzia* is a short-chain fatty acid-producing bacteria. Supplementation with *Adlercreutzia equolifaciens* significantly lowered blood pressure in rats resistant to antihypertensive drugs.^83^ *Phascolarctobacterium* accumulated in people with mild cognitive impairment or Alzheimer’s disease.^84^ On the other hand, *Phascolarctobacterium* is also a short-chain fatty acid-producing bacteria that improves lipid metabolism and insulin sensitivity.^85^ Intestinal *Collinsella* was found to be correlated with nonalcoholic steato-hepatitis in humans.^86^ *Collinsella*, which produces butyric acid, was found to be significantly reduced in newly diagnosed patients with acute myeloid leukemia.^87^ *Streptococcus*, known as another genus of short-chain fatty acid-producing bacteria, functions as a probiotic in the alleviation of gastroenteritis.^88^ Nevertheless, some *Streptococcus* species, such as *Streptococcus gallolyticus* subsp. *Gallolyticus* are bacterial pathogens involved in bacteremia and endocarditis and are usually associated with colon tumors in elderly patients.^89^

## Contradictory reports of microplastics' effects on some bacteria

Lachnospiraceae_NK4A136_group (from the Lachnospiraceae family) was also found to be negatively associated with the level of MPs in meconium samples^90^ and the intestinal tracts of preschool children.^70^ Decreased abundance of Lachnospiraceae was confirmed to be correlated with the onset of IBD.^71^ However, a contradictory report with postgraduate students indicated that *Dorea* (a genus in Lachnospiraceae) was positively associated with consuming takeaway foods (Figure 1).^62^ *Dorea* was more abundant in the intestines of patients with either nonalcoholic steatohepatitis or nonalcoholic fatty liver disease, implying that it may be a pathobiont.^91^ Differences in host age may be a potential reason for this contradiction.

Hong et al.^68^ reported that *Alistipes* increased in young fatty liver disease patients. In contrast, in a pilot study of preschool children, MP exposure was negatively correlated with *Alistipes* (Figure 1).^70^ A high-fat diet has been reported to increase *Alistipes* sp. *Marseille-P5997* and *Alistipes* sp. *5CPEGH6* in mouse intestines.^92^ *Alistipes shahii* and *Alistipes indistinctus* have been found to be positively associated with obesity in humans.^93^ Nevertheless, *Alistipes finegoldii* has also been deemed a probiotic that may protect hosts against colitis and IBS.^94^ For young adults, hot foods served in disposable plastic tableware are considered potential sources of MPs.^68^ However, for preschool children, breastmilk and formula milk in plastic bottles are considered potential sources of MPs.^70^ Different food containers release distinct MPs. Consequently, in addition to host factors (e.g., age and diet), MP characteristics (type, size, shape) may influence specific bacterial abundances.

Most studies with adults have indicated that MPs are negatively correlated with *Faecalibacterium*.^51^^,^^62^^,^^68^^,^^95^ Ke et al.^70^ reported that *Faecalibacterium* increased in preschool children (Figure 1). *Faecalibacterium* is a genus of acid-producing bacteria that is abundant in the human intestine. Its population correlates with multiple inflammatory diseases, such as IBS and IBD.^96^ A previous study found that *Faecalibacterium prausnitzii* abundance was significantly reduced in the guts of patients with Crohn’s disease.^97^ However, two other studies demonstrated that *Faecalibacterium prausnitzii* was increased in children with Crohn’s disease, implying that *Faecalibacterium* may be detrimental, at least in pediatric Crohn’s disease,^98^^,^^99^ which is consistent with the positive correlation between MPs and *Faecalibacterium* being found only in preschool children.^70^

Given the functional heterogeneity observed at both the species and strain levels, future research on the human gut microbiome should prioritize deeper taxonomic resolution. Specifically, investigations into Lachnospiraceae should advance to the species level, while analyses of genera such as *Alistipes* and *Faecalibacterium* should strive for strain-level characterization. To achieve this, shotgun metagenomic sequencing is recommended over 16S meta-barcoding, as it provides superior power for identifying less abundant taxa and enables higher-resolution profiling.^100^ Furthermore, it is crucial to strictly control for key confounders such as diet, lifestyle, and medication. For instance, gut microbiome data from children and adults should not be directly compared without accounting for age-related compositional differences.

## Association between microplastic-induced gut microbiome dysbiosis and human diseases

There is also limited evidence of human diseases resulting from MP exposure. In a study with college students aged 18−30 years, the body fat percentage, visceral fat area, and body mass index (BMI) of the high-MP exposure group were significantly higher than those of the low-MP exposure group, where *Veillonella*, *Alistipes*, *Turicibacter*, Rikenellaceae, and *Eubacterium coprostanoligenes* may be involved in the occurrence of obesity.^68^ Another report demonstrated that the participants, who consumed takeaway foods in disposable plastic containers, had a higher prevalence of gastrointestinal dysfunction and cough than did the control group, where the gut *Faecalibacterium* was most strongly correlated with occasional consumers and the gut *Collinsella* was most strongly correlated with frequent consumers (Table 1).^101^ However, we cannot exclude the possibility that takeaway foods may contain pathobionts or some gastrointestinal irritants. More rigorously controlled experimental designs are needed. Both studies enrolled young adults. Considering that children are more sensitive to MPs than adults are,^57^^,^^70^ more gut microbiome-coupled studies in children are warranted.

**Table 1.** Human diseases associated with microplastics and the coupled gut microbiome studies.

Subjects	Associated diseases	Gut microbiome	References
College students aged 18–30 years	Obesity	studied	[68]
Students aged 18–30 years	Gastrointestinal dysfunction and cough	studied	[101]
Sixty-one tumor samples	Lung, cervical, gastric, colorectal and pancreatic cancer	Not studied	[102]
Colorectal cancer patients	Colorectal cancer	Not studied	[103–105]
IBD patients	Inflammatory bowel disease	Not studied	[106]
Crohn's disease patients	Fibrotic intestines	Not studied	[107]

MPs are detected in 70% of pancreatic tumors, as well as in gastric, lung, colorectal, and cervical tumors, but are not detected in esophageal tumors.^102^ MPs have also been detected in tumoral and peritumoral tissues of patients with colorectal cancer (Table 1).^103-105^ However, associations between MP exposure and cancer may not be attributed to gut microbiota dysbiosis. Tumors and normal tissues have different affinities for MPs. Some molecules on the tumor cell surface exhibit high affinities for various MPs. One study suggested that MPs selectively bind to the cell surface receptor T-cell membrane protein 4 (TIM-4), promoting the entry of MPs into the cell.^108^ The role of the gut microbiota in carcinogenesis induced by MP exposure needs further investigation.

Yan et al.^106^ reported a positive correlation between the level of stool MPs and the severity of IBD (Table 1). They suggested that MP exposure may be correlated with the disease process or that IBD may aggravate the retention of MPs.^106^ However, gut microbiome studies in IBD patients exposed to MPs are still lacking.^109^

In a study with Crohn's disease patients, MPs levels were positively associated with the severity of intestinal fibrosis (Table 1).^107^ Some high-risk practices, e.g. frequently invasive gastrointestinal tract examinations, aggravated MPs accumulation in fibrotic intestines.^107^ And significant increases in MPs levels at the lesion sites were observed when comparing with the surrounding tissues.^107^ In a mouse model with chronic colitis, MPs led to higher prevalence of all pathological changes in general, and ulcers in particular, in a greater number of crypt abscesses and enteroendocrine cells.^110^ Therefore, MPs, as well as their correlated gut microbiota dysbiosis, may play different roles between ulcerative colitis and non-ulcerative colitis.

Exposure to MPs led to an increase in depression-associated microbiota (such as Actinomycetota, Pseudomonadota, Bacillota, and Bacteroidota phyla).^62^^,^^68^ In mice with Parkinson's disease, MPs exacerbated the spread of *α*-synuclein pathology across dopaminergic neurons in the substantia nigra.^111^ Considering the importance of the brain-gut axis, correlations between mental sub-health status and gut microbiome in people with high MPs exposure should be explored.

Mouse experiments demonstrated that gut microbiota contributed to MPs-induced colonic and hepatic injuries.^9^ Extended exposure to MPs caused inflammatory damages to the liver by a mechanism that imbalance in intestinal flora was crucial for MPs-induced liver pyroptosis.^112^ Aged MPs led to more severe hepatic dysfunction and gut microbiota dysbiosis.^113^ This dysregulation may be mediated by the fatty acid signaling derived hepatic lipolysis disorders and oxidative damages.^113^ All these reports highlighted a connection between MPs and the gut-liver axis.^114^ However, liver diseases in humans exposed to MPs have not been well assessed.

## Microplastics affect multiple organ microbiota

In the individuals consuming take-away food in disposable plastic containers, the gut microbiota was associated with oral microbial abundance and evenness, implying that the diversity of oral microbiota can be influenced by the intestinal bacteria in this cohort.^101^ Zhang et al.^115^ recruited 20 participants from a plastic factory (high MPs exposure area) and the other 20 participants from a park (low MPs exposure area). Their analysis revealed that high MP exposure not only led to changes in the dominant gut microbiota and nasal microbiota but also altered their symbiotic relationship with each other.^115^ MPs have also been found in human placental and meconium samples, suggesting that the wide exposure of pregnant women and infants.^90^ Moreover, a correlation between MP levels and microbiota genera in the placenta and meconium has been found.^90^ More correlation analyses of multiple organ microbiota in people with high MP exposure should be carried out.

## Critical limitations in current research on microplastics and the gut microbiome

While a growing body of evidence suggests that MPs can alter the gut microbiome and contribute to adverse health outcomes, the interpretation of this literature is constrained by several significant methodological challenges. Key limitations pertain to exposure assessment, the resolution of microbiome analysis, and controlling for critical confounding variables.

A fundamental challenge in this field is the accurate quantification and characterization of human MP exposure. Many studies rely on in vitro models or animal experiments using standardized, spherical MP polymers (e.g., polystyrene beads) at high concentrations.^15^ While controlled, these conditions do not reflect the complex reality of human exposure, which involves a heterogeneous mixture of particles varying in polymer type, size, shape, and surface chemistry, often with copollutants such as additives or adsorbed pathogens.^17^ Furthermore, extrapolating doses from animal studies to human-relevant exposures remains problematic. In human studies, exposure is often inferred indirectly from environmental data or food packaging usage, lacking direct measurement of the internal dose.^1^ The absence of standardized, sensitive, and cost-effective methods for detecting and quantifying MPs in human tissues and feces continues to hinder our ability to establish robust dose‒response relationships.

The choice of analytical technique profoundly impacts the conclusions drawn about MP-induced microbial shifts. Most current studies utilize 16S rRNA gene amplicon sequencing, which is cost effective for profiling microbial community structure at the genus level. However, this method has limited taxonomic resolution, often failing to distinguish species- or strain-level changes that are critical for understanding functional shifts, such as the bloom of a specific pathogenic strain versus a benign one within the same genus.^100^ More importantly, 16S data infer functional potential only indirectly. In contrast, shotgun metagenomic sequencing provides a comprehensive view of the entire genetic repertoire, allowing direct inference of microbial functions,^100^ such as antibiotic resistance genes, virulence factors, and pathways for xenobiotic degradation that may be activated by MP exposure. The reliance on 16S rRNA gene amplicon sequencing in many studies thus likely obscures the most functionally relevant impacts of MPs on the gut microbiome.

The human gut microbiome is highly dynamic and influenced by a multitude of factors that can confound or obscure the effects of MP exposure. Key among these factors are diet, lifestyle, and medication use. For instance, hot foods served in disposable plastic tableware are a major source of MP exposure and independently drive dysbiosis, creating a tangled web of association.^62^^,^^68^^,^^101^ Similarly, the use of proton pump inhibitors or antibiotics drastically remodels the gut microbiota,^116^^,^^117^ an effect that could be misattributed to MP coexposure. Many epidemiological and even some interventional studies lack the granular, high-quality data required to adequately control for these powerful confounders. Failure to meticulously account for these variables through rigorous study design and sophisticated statistical models introduces substantial uncertainty and complicates the isolation of the unique effect size attributable to MPs themselves.

## Conclusions

Current research on the impact of microplastics (MPs) on the human gut microbiota faces significant limitations, particularly in exposure assessment. Future studies must prioritize the development of validated MP biomonitoring methods, the adoption of high-resolution shotgun metagenomics, and the implementation of longitudinal designs that rigorously control for confounders such as diet and medication. Only by addressing these challenges can we progress from observing associations to establishing a causal role for MPs in human health.

Indeed, MP-induced dysbiosis has been correlated with several conditions, including obesity, gastrointestinal disorders, inflammatory bowel disease, and certain cancers. In addition, MP exposure has been linked to an increase in depression-associated microbiota, highlighting the need to explore the gut‒brain axis in populations with high MP exposure. Furthermore, the gut-liver axis represents another critical pathway; the role of the gut microbiome in MP-associated liver diseases warrants further investigation in human populations.

## Data Availability

Data sharing is not applicable to this article as no new data were created or analyzed in this review.

## References

[1] Zhao H, Lin G, Yin Y, Wu Q, Wang Y, Tang N, Qi X. Impact of micro- and nanoplastics on gastrointestinal diseases: recent advances. Eur J Intern Med. 2025;139(1):106419. doi: 10.1016/j.ejim.2025.07.015. 40701881

[2] Li Y, Tao L, Wang Q, Wang F, Li G, Song M. Potential health impact of microplastics: a review of environmental distribution, human exposure, and toxic effects. Environ Health. 2023;1(4):249–257. doi: 10.1021/envhealth.3c00052. PMC11504192 39474495

[3] Sorci G, Loiseau C. Should we worry about the accumulation of microplastics in human organs? EBioMedicine. 2022;82(1):104191. doi: 10.1016/j.ebiom.2022.104191. 35907367 PMC9335379

[4] Abbasi S, Turner A. Human exposure to microplastics: a study in Iran. J Hazard Mater. 2021;403(1):123799. doi: 10.1016/j.jhazmat.2020.123799. 33264903

[5] Huang S, Huang X, Bi R, Guo Q, Yu X, Zeng Q, Huang Z, Liu T, Wu H, Chen Y, et al. Detection and analysis of microplastics in human sputum. Environ Sci Technol. 2022;56(4):2476–2486. doi: 10.1021/acs.est.1c03859. 35073488

[6] Leslie HA, van Velzen MJM, Brandsma SH, Vethaak AD, Garcia-Vallejo JJ, Lamoree MH. Discovery and quantification of plastic particle pollution in human blood. Environ Int. 2022;163(1):107199. doi: 10.1016/j.envint.2022.107199. 35367073

[7] Horvatits T, Tamminga M, Liu B, Sebode M, Carambia A, Fischer L, Püschel K, Huber S, Fischer EK. Microplastics detected in cirrhotic liver tissue. EBioMedicine. 2022;82(1):104147. doi: 10.1016/j.ebiom.2022.104147. 35835713 PMC9386716

[8] Thompson RC, Courtene-Jones W, Boucher J, Pahl S, Raubenheimer K, Koelmans AA. Twenty years of microplastic pollution research-what have we learned? Sci. 2024;386(6720):eadl2746. doi: 10.1126/science.adl2746. 39298564

[9] Zhang K, Yang J, Chen L, He J, Qu D, Zhang Z, Liu Y, Li X, Liu J, Li J, et al. Gut microbiota participates in polystyrene microplastics-induced hepatic injuries by modulating the gut-liver axis. ACS Nano. 2023;17(15):15125–15145. doi: 10.1021/acsnano.3c04449. 37486121

[10] Zhang Z, Chen W, Chan H, Peng J, Zhu P, Li J, Jiang X, Zhang Z, Wang Y, Tan Z, et al. Polystyrene microplastics induce size-dependent multi-organ damage in mice: insights into gut microbiota and fecal metabolites. J Hazard Mater. 2024;461(1):132503. doi: 10.1016/j.jhazmat.2023.132503. 37717443

[11] Wang J, Yang Y, Shi Y, Wei L, Gao L, Liu M. Oxidized/unmodified-polyethylene microplastics neurotoxicity in mice: perspective from microbiota-gut-brain axis. Environ Int. 2024;185(1):108523. doi: 10.1016/j.envint.2024.108523. 38484610

[12] Bao L, Cui X, Zeng T, Liu G, Lai W, Zhao H, Gao F, Wu J, Leong KW, Chen C. Incorporation of polylactic acid microplastics into the carbon cycle as a carbon source to remodel the endogenous metabolism of the gut. Proc Natl Acad Sci U S A. 2025;122(19):e2417104122. doi: 10.1073/pnas.2417104122. 40324088 PMC12088454

[13] Xu Y, Ou Q, Wang X, Hou F, Li P, van der Hoek JP, Liu G. Assessing the mass concentration of microplastics and nanoplastics in wastewater treatment plants by pyrolysis gas chromatography-mass spectrometry. Environ Sci Technol. 2023;57(8):3114–3123. doi: 10.1021/acs.est.2c07810. 36787182 PMC9979646

[14] Barbosa F, Adeyemi JA, Bocato MZ, Comas A, Campiglia A. A critical viewpoint on current issues, limitations, and future research needs on micro- and nanoplastic studies: from the detection to the toxicological assessment. Environ Res. 2020;182(1):109089. doi: 10.1016/j.envres.2019.109089. 32069751

[15] Park JS, Yoo JW, Lee YH, Park C, Lee YM. Size- and shape-dependent ingestion and acute toxicity of fragmented and spherical microplastics in the absence and presence of prey on two marine zooplankton. Mar Pollut Bull. 2024;206(1):116768. doi: 10.1016/j.marpolbul.2024.116768. 39067234

[16] Rong J, Yuan C, Yin X, Wu X, He F, Wang Y, Leung KS, Lin S. Co-exposure of polystyrene nanoplastics and copper induces development toxicity and intestinal mitochondrial dysfunction in vivo and in vitro. Sci Total Environ. 2024;930(1):172681. doi: 10.1016/j.scitotenv.2024.172681. 38663618

[17] Demarquoy J. Microplastics and probiotics: mechanisms of interaction and their consequences for health. AIMS Microbiol. 2025;11(2):388–409. doi: 10.3934/microbiol.2025018. 40600211 PMC12207257

[18] Bao X, Gu Y, Chen L, Wang Z, Pan H, Huang S, Meng Z, Chen X. Microplastics derived from plastic mulch films and their carrier function effect on the environmental risk of pesticides. Sci Total Environ. 2024;924:171472. doi: 10.1016/j.scitotenv.2024.171472. 38458459

[19] Stenger KS, Wikmark OG, Bezuidenhout CC, Molale-Tom LG. Microplastics pollution in the ocean: potential carrier of resistant bacteria and resistance genes. Environ Pollut. 2021;291:118130. doi: 10.1016/j.envpol.2021.118130. 34562691

[20] Groschwitz KR, Hogan SP. Intestinal barrier function: molecular regulation and disease pathogenesis. J Allergy Clin Immunol. 2009;124:3–20. doi: 10.1016/j.jaci.2009.05.038. 19560575 PMC4266989

[21] Liang B, Zhong Y, Huang Y, Lin X, Liu J, Lin L, Hu M, Jiang J, Dai M, Wang B, et al. Underestimated health risks: polystyrene micro- and nanoplastics jointly induce intestinal barrier dysfunction by ROS-mediated epithelial cell apoptosis. Part Fibre Toxicol. 2021;18(1):20. doi: 10.1186/s12989-021-00414-1. 34098985 PMC8186235

[22] Deng Y, Yan Z, Shen R, Wang M, Huang Y, Ren H, Zhang Y, Lemos B. Microplastics release phthalate esters and cause aggravated adverse effects in the mouse gut. Environ Int. 2020;143:105916. doi: 10.1016/j.envint.2020.105916. 32615348

[23] Han S, Bang J, Choi D, Hwang J, Kim T, Oh Y, Hwang Y, Choi J, Hong J. Surface pattern analysis of microplastics and their impact on human-derived cells. ACS Appl Polym Mater. 2020;2:4541–4550. doi: 10.1021/acsapm.0c00645.

[24] Li B, Ding Y, Cheng X, Sheng D, Xu Z, Rong Q, Wu Y, Zhao H, Ji X, Zhang Y. Polyethylene microplastics affect the distribution of gut microbiota and inflammation development in mice. Chsph. 2020;244:125492. doi: 10.1016/j.chemosphere.2019.125492. 31809927

[25] Zeng G, Li J, Wang Y, Su J, Lu Z, Zhang F, Ding W. Polystyrene microplastic-induced oxidative stress triggers intestinal barrier dysfunction via the NF-κb/NLRP3/IL- 1β/MCLK pathway. Environ Pollut. 2024;345:123473. doi: 10.1016/j.envpol.2024.123473. 38301820

[26] Kim DH, Lee S, Ahn J, Kim JH, Lee E, Lee I, Byun S. Transcriptomic and metabolomic analysis unveils nanoplastic-induced gut barrier dysfunction via STAT1/6 and ERK pathways. Environ Res. 2024;249:118437. doi: 10.1016/j.envres.2024.118437. 38346486

[27] Su QL, Wu J, Tan SW, Guo XY, Zou DZ, Kang K. The impact of microplastics polystyrene on the microscopic structure of mouse intestine, tight junction genes and gut microbiota. PLoS One. 2024;19(6):e0304686. doi: 10.1371/journal.pone.0304686. 38837998 PMC11152276

[28] Jin Y, Lu L, Tu W, Luo T, Fu Z. Impacts of polystyrene microplastic on the gut barrier, microbiota and metabolism of mice. Sci Total Environ. 2019;649:308–317. doi: 10.1016/j.scitotenv.2018.08.353. 30176444

[29] Qiao J, Chen R, Wang M, Bai R, Cui X, Liu Y, Wu C, Chen C. Perturbation of gut microbiota plays an important role in micro/nanoplastics-induced gut barrier dysfunction. Nanoscale. 2021;13:8806–8816. doi: 10.1039/d1nr00038a. 33904557

[30] Galloway TS, Cole M, Lewis C. Interactions of microplastic debris throughout the marine ecosystem. Nat Ecol Evol. 2017;1(5):116. doi: 10.1038/s41559-017-0116. 28812686

[31] Nosyk O, ter Haseborg E, Metzger U, Frimmel FH. A standardized pre-treatment method of biofilm flocs for fluorescence microscopic characterization. J Microbiol Methods. 2008;75(3):449–456. doi: 10.1016/j.mimet.2008.07.024. 18718852

[32] Rogers J, Dowsett A, Dennis P, Lee J, Keevil C. Influence of plumbing materials on biofilm formation and growth of *Legionella pneumophila* in potable water systems. Appl Environ Microbiol. 1994;60(6):1842–1851. doi: 10.1128/aem.60.6.1842-1851.1994. 16349278 PMC201571

[33] Santana M, Ascer L, Custódio M, Moreira F, Turra A. Microplastic contamination in natural mussel beds from a Brazilian urbanized coastal region: rapid evaluation through bioassessment. Mar Pollut Bull. 2016;106(1-2):183–189. doi: 10.1016/j.marpolbul.2016.02.074. 26980138

[34] Zhang B, Yang X, Liu L, Chen L, Teng J, Zhu X, Zhao J, Wang Q. Spatial and seasonal variations in biofilm formation on microplastics in coastal waters. Sci Total Environ. 2021;770:145303. doi: 10.1016/j.scitotenv.2021.145303. 33515883

[35] Kesy K, Oberbeckmann S, Kreikemeyer B, Labrenz M. Spatial environmental heterogeneity determines young biofilm assemblages on microplastics in Baltic Sea mesocosms. Front Microbiol. 2019;10:1665. doi: 10.3389/fmicb.2019.01665. 31447791 PMC6696623

[36] Mittal N, Tiwari N, Singh D, Tripathi P, Sharma S. Toxicological impacts of microplastics on human health: a bibliometric analysis. Environ Sci Pollut Res Int. 2024;31(46):57417–57429. doi: 10.1007/s11356-023-30801-4. 37936032

[37] Jiménez-Arroyo C, Tamargo A, Molinero N, Reinosa JJ, Alcolea-Rodriguez V, Portela R, Bañares MA, Fernández JF, Moreno-Arribas MV. Simulated gastrointestinal digestion of polylactic acid (PLA) biodegradable microplastics and their interaction with the gut microbiota. Sci Total Environ. 2023;902:166003. doi: 10.1016/j.scitotenv.2023.166003. 37549707

[38] Bakiera A, Solarz A, Kowalczyk M, Cichoż-Lach H, Korona-Głowniak I. Challenges and prospects for eradication of *Helicobacter pylori*: targeting virulence factors, metabolism, and vaccine innovation. Pathogens. 2025;14(7):619. doi: 10.3390/pathogens14070619. 40732667 PMC12299007

[39] Cai L, Wang J, Peng J, Wu Z, Tan X. Observation of the degradation of three types of plastic pellets exposed to UV irradiation in three different environments. Sci Total Environ. 2018;628-629:740–747. doi: 10.1016/j.scitotenv.2018.02.079. 29454214

[40] Khoironi A, Hadiyanto H, Anggoro S, Sudarno S. Evaluation of polypropylene plastic degradation and microplastic identification in sediments at Tambak Lorok coastal area, Semarang, Indonesia. Mar Pollut Bull. 2020;151:110868. doi: 10.1016/j.marpolbul.2019.110868. 32056648

[41] Stock V, Fahrenson C, Thuenemann A, Dönmez MH, Voss L, Böhmert L, Braeuning A, Lampen A, Sieg H. Impact of artificial digestion on the sizes and shapes of microplastic particles. Food Chem Toxicol. 2020;135:111010. doi: 10.1016/j.fct.2019.111010. 31794801

[42] Zhang K, Hamidian AH, Tubić A, Zhang Y, Fang JKH, Wu C, Lam PKS. Understanding plastic degradation and microplastic formation in the environment: a review. Environ Pollut. 2021;274:116554. doi: 10.1016/j.envpol.2021.116554. 33529891

[43] Kirstein IV, Kirmizi S, Wichels A, Garin-Fernandez A, Erler R, Löder M, Gerdts G. Dangerous hitchhikers? Evidence for potentially pathogenic *Vibrio* spp. on microplastic particles. Mar Environ Res. 2016;120:1–8. doi: 10.1016/j.marenvres.2016.07.004. 27411093

[44] Beloe CJ, Browne MA, Johnston EL. Plastic debris as a vector for bacterial disease: an interdisciplinary systematic review. Environ Sci Technol. 2022;56(5):2950–2958. doi: 10.1021/acs.est.1c05405. 35129968

[45] Bowley J, Baker-Austin C, Porter A, Hartnell R, Lewis C. Oceanic hitchhikers - assessing pathogen risks from marine microplastic. Trends Microbiol. 2021;29(2):107–116. doi: 10.1016/j.tim.2020.06.011. 32800610

[46] Wu X, Pan J, Li M, Li Y, Bartlam M, Wang Y. Selective enrichment of bacterial pathogens by microplastic biofilm. Water Res. 2019;165:114979. doi: 10.1016/j.watres.2019.114979. 31445309

[47] Baral B, Kashyap D, Varshney N, Verma TP, Jain AK, Chatterji D, Kumar V, Mishra A, Kumar A, Jha HC. Data on differential pathogenic ability of *Helicobacter pylori* isolated from distinct gastric niches. Data Brief. 2023;47:108981. doi: 10.1016/j.dib.2023.108981. 36875222 PMC9975699

[48] Gobert AP, Wilson KT. Induction and regulation of the innate immune response in *Helicobacter pylori* infection. Cell Mol Gastroenterol Hepatol. 2022;13(5):1347–1363. doi: 10.1016/j.jcmgh.2022.01.022. 35124288 PMC8933844

[49] Kandpal M, Indari O, Baral B, Jakhmola S, Tiwari D, Bhandari V, Pandey RK, Bala K, Sonawane A, Jha HC. Dysbiosis of gut microbiota from the perspective of the gut-brain axis: role in the provocation of neurological disorders. Metabolites. 2022;12(11):1064. doi: 10.3390/metabo12111064. 36355147 PMC9692419

[50] Krzyżek P, Grande R, Migdał P, Paluch E, Gościniak G. Biofilm formation as a complex result of virulence and adaptive responses of *Helicobacter pylori*. Pathogens. 2020;9(12):1062. doi: 10.3390/pathogens9121062. 33353223 PMC7766044

[51] Gao B, Chen L, Wu L, Zhang S, Zhao S, Mo Z, Chen Z, Tu P. Association between microplastics and the functionalities of human gut microbiome. Ecotoxicol Environ Saf. 2025;290(1):117497. doi: 10.1016/j.ecoenv.2024.117497. 39708450

[52] Choi YJ, Kim JE, Lee SJ, Gong JE, Jin YJ, Seo S, Lee JH, Hwang DY. Inflammatory response in the mid colon of ICR mice treated with polystyrene microplastics for two weeks. Lab Anim Res. 2021;37(1):31. doi: 10.1186/s42826-021-00109-w. 34809705 PMC8607556

[53] Yang Q, Dai H, Wang B, Xu J, Zhang Y, Chen Y, Ma Q, Xu F, Cheng H, Sun D, et al. Nanoplastics shape adaptive anticancer immunity in the colon in mice. Nano Lett. 2023;23(8):3516–3523. doi: 10.1021/acs.nanolett.3c00644. 37043775

[54] Wang H, Xu K, Wang J, Feng C, Chen Y, Shi J, Ding Y, Deng C, Liu X. Microplastic biofilm: an important microniche that may accelerate the spread of antibiotic resistance genes via natural transformation. J Hazard Mater. 2023;459:132085. doi: 10.1016/j.jhazmat.2023.132085. 37494793

[55] Wang J, Wang Y, Li Z, Wang J, Zhao H, Zhang X. Gut microbiota, a key to understanding the knowledge gaps on micro-nanoplastics-related biological effects and biodegradation. Sci Total Environ. 2024;944:173799. doi: 10.1016/j.scitotenv.2024.173799. 38852863

[56] Sinha P, Saini V, Varshney N, Pandey RK, Jha HC. The infiltration of microplastics in human systems: gastrointestinal accumulation and pathogenic impacts. Heliyon. 2025;11(4):e42606. doi: 10.1016/j.heliyon.2025.e42606. 40061927 PMC11889576

[57] Fournier E, Ratel J, Denis S, Leveque M, Ruiz P, Mazal C, Amiard F, Edely M, Bezirard V, Gaultier E, et al. Exposure to polyethylene microplastics alters immature gut microbiome in an infant in vitro gut model. J Hazard Mater. 2023;443(Pt B):130383. doi: 10.1016/j.jhazmat.2022.130383. 36444070

[58] Liu W, Zhang R, Shu R, Yu J, Li H, Long H, Jin S, Li S, Hu Q, Yao F, et al. Study of the relationship between microbiome and colorectal cancer susceptibility using 16SrRNA sequencing. BioMed Res Int. 2020;2020(1):7828392. doi: 10.1155/2020/7828392. 32083132 PMC7011317

[59] Pittayanon R, Lau JT, Yuan Y, Leontiadis GI, Tse F, Surette M, Moayyedi P. Gut microbiota in patients with irritable bowel syndrome - A systematic review. Gastroenterology. 2019;157(1):97–108. doi: 10.1053/j.gastro.2019.03.049. 30940523

[60] Zhu BK, Fang YM, Zhu D, Christie P, Ke X, Zhu YG. Exposure to nanoplastics disturbs the gut microbiome in the soil oligochaete *Enchytraeus crypticus*. Environ Pollut. 2018;239(1):408–415. doi: 10.1016/j.envpol.2018.04.017. 29679938

[61] de Vries SP, Bootsma HJ, Hays JP, Hermans PW. Molecular aspects of *Moraxella catarrhalis* pathogenesis. Microbiol Mol Biol Rev. 2009;73(3):389–406. doi: 10.1128/MMBR.00007-09. 19721084 PMC2738133

[62] Zhang X, He X, Pan D, Shi L, Wu Y, Yang Y, Zhu Y, Wang Y, Wang H, Pu L, et al. Effects of thermal exposure to disposable plastic tableware on human gut microbiota and metabolites: A quasi-experimental study. J Hazard Mater. 2024;462(1):132800. doi: 10.1016/j.jhazmat.2023.132800. 37866144

[63] Oren A. On validly published names, correct names, and changes in the nomenclature of phyla and genera of prokaryotes: a guide for the perplexed. NPJ Biofilms Microbiomes. 2024;10(1):20. doi: 10.1038/s41522-024-00494-9. 38467688 PMC10928132

[64] Li D, Sun T, Tong Y, Le J, Yao Q, Tao J, Liu H, Jiao W, Mei Y, Chen J, et al. Gut-microbiome-expressed 3β-hydroxysteroid dehydrogenase degrades estradiol and is linked to depression in premenopausal females. Cell Metab. 2023;35(4):685–694. doi: 10.1016/j.cmet.2023.02.017. 36933555

[65] Bergstrand LH, Cardenas E, Holert J, Van Hamme JD, Mohn WW. Delineation of steroid-degrading microorganisms through comparative genomic analysis. mBio. 2016;7(2):e00166. doi: 10.1128/mBio.00166-16. 26956583 PMC4810484

[66] Wang T, Sha L, Li Y, Zhu L, Wang Z, Li K, Lu H, Bao T, Guo L, Zhang X, et al. Dietary α-linolenic acid-rich flaxseed oil exerts beneficial effects on polycystic ovary syndrome through sex steroid hormones-microbiota- inflammation axis in rats. Front Endocrinol. 2020;11(1):284. doi: 10.3389/fendo.2020.00284. PMC7326049 32670195

[67] Morssinkhof MWL, van Wylick DW, Priester-Vink S, van der Werf YD, den Heijer M, van den Heuvel OA, Broekman BFP. Associations between sex hormones, sleep problems and depression: a systematic review. Neurosci Biobehav Rev. 2020;118(1):669–680. doi: 10.1016/j.neubiorev.2020.08.006. 32882313

[68] Hong Y, Feng Y, Yan T, Zhang L, Zhao Q, Zhao Q, Huang J, Huang S, Zhang Y. Take-out food enhances the risk of MPs ingestion and obesity, altering the gut microbiome in young adults. J Hazard Mater. 2024;476(1):135125. doi: 10.1016/j.jhazmat.2024.135125. 39003809

[69] Mayneris-Perxachs J, Cardellini M, Hoyles L, Latorre J, Davato F, Moreno-Navarrete JM, Arnoriaga-Rodríguez M, Serino M, Abbott J, Barton RH, et al. Iron status influences non-alcoholic fatty liver disease in obesity through the gut microbiome. Microbiome. 2021;9(1):104. doi: 10.1186/s40168-021-01052-7. 33962692 PMC8106161

[70] Ke D, Zheng J, Liu X, Xu X, Zhao L, Gu Y, Yang R, Liu S, Yang S, Du J, et al. Occurrence of microplastics and disturbance of gut microbiota: a pilot study of preschool children in Xiamen, China. EBioMedicine. 2023;97(1):104828. doi: 10.1016/j.ebiom.2023.104828. 37837933 PMC10585208

[71] Noor SO, Ridgway K, Scovell L, Kemsley EK, Lund EK, Jamieson C, Johnson IT, Narbad A. Ulcerative colitis and irritable bowel patients exhibit distinct abnormalities of the gut microbiota. BMC Gastroenterol. 2010;10(1):134. doi: 10.1186/1471-230X-10-134. 21073731 PMC3002299

[72] Maukonen J, Kolho KL, Paasela M, Honkanen J, Klemetti P, Vaarala O, Saarela M. Altered fecal microbiota in paediatric inflammatory bowel disease. J Crohns Colitis. 2015;9(12):1088–1095. doi: 10.1093/ecco-jcc/jjv147. 26351391

[73] Wei W, Jiang W, Tian Z, Wu H, Ning H, Yan G, Zhang Z, Li Z, Dong F, Sun Y, et al. Fecal *g. Streptococcus* and *g. Eubacterium_coprostanoligenes_group* combined with sphingosine to modulate the serum dyslipidemia in high-fat diet mice. Clin Nutr. 2021;40(6):4234–4245. doi: 10.1016/j.clnu.2021.01.031. 33608131

[74] Zhang X, Wang S, Xu H, Yi H, Guan J, Yin S. Metabolomics and microbiome profiling as biomarkers in obstructive sleep apnoea: a comprehensive review. Eur Respir Rev. 2021;30(160):200220. doi: 10.1183/16000617.0220-2020. 33980666 PMC9489097

[75] Matenchuk BA, Mandhane PJ, Kozyrskyj AL. Sleep, circadian rhythm, and gut microbiota. Sleep Med Rev. 2020;53(1):101340. doi: 10.1016/j.smrv.2020.101340. 32668369

[76] Saedi S, Derakhshan S, Sadeghi J, Hasani A, Khoshbaten M, Poortahmasebi V, Ahmadi S. A study on gut microbiota and short-chain fatty acids in patients with inflammatory bowel disease from northwest Iran. Lett Appl Microbiol. 2025;78(8):ovaf111. doi: 10.1093/lambio/ovaf111. 40802486

[77] Nie K, Ma K, Luo W, Shen Z, Yang Z, Xiao M, Tong T, Yang Y, Wang X. *Roseburia intestinalis*: a beneficial gut organism from the discoveries in genus and species. Front Cell Infect Microbiol. 2021;11(1):757718. doi: 10.3389/fcimb.2021.757718. 34881193 PMC8647967

[78] Mandal S, Van Treuren W, White RA, Eggesbø M, Knight R, Peddada SD. Analysis of composition of microbiomes: a novel method for studying microbial composition. Microb Ecol Health Dis. 2015;26(1):27663. doi: 10.3402/mehd.v26.27663. 26028277 PMC4450248

[79] Balaich J, Estrella M, Wu G, Jeffrey PD, Biswas A, Zhao L, Korennykh A, Donia MS. The human microbiome encodes resistance to the antidiabetic drug acarbose. Nature. 2021;600(7887):110–115. doi: 10.1038/s41586-021-04091-0. 34819672 PMC10258454

[80] Raygoza Garay JA, Turpin W, Lee SH, Smith MI, Goethel A, Griffiths AM, Moayyedi P, Espin-Garcia O, Abreu M, Aumais GL, et al. Gut microbiome composition is associated with future onset of Crohn's disease in healthy first-degree relatives. Gastroenterology. 2023;165(3):670–681. doi: 10.1053/j.gastro.2023.05.032. 37263307

[81] Yan F, Zhang Q, Shi K, Zhang Y, Zhu B, Bi Y, Wang X. Gut microbiota dysbiosis with hepatitis B virus liver disease and association with immune response. Front Cell Infect Microbiol. 2023;13(1):1152987. doi: 10.3389/fcimb.2023.1152987. 37201112 PMC10185817

[82] Wang M, Amakye WK, Gong C, Ren Z, Yuan E, Ren J. Effect of oral and intraperitoneal administration of walnut-derived pentapeptide PW5 on cognitive impairments in APP_SWE_/PS1_ΔE9_ mice. Free Radic Biol Med. 2022;180(1):191–197. doi: 10.1016/j.freeradbiomed.2022.01.003. 35077820

[83] Yun F, Han X, Wang Z, Gao Q, Xu M, Liu H, Fang N, Zhang Y, Li Y, Gong Y. Intermittent fasting ameliorates resistant hypertension through modulation of gut microbiota. Pharmacol Res. 2025;219(1):107864. doi: 10.1016/j.phrs.2025.107864. 40701347

[84] Son SJ, Wu X, Roh HW, Cho YH, Hong S, Nam YJ, Hong CH, Park S. Distinct gut microbiota profiles and network properties in older Korean individuals with subjective cognitive decline, mild cognitive impairment, and Alzheimer's disease. Alzheimers Res Ther. 2025;17(1):187. doi: 10.1186/s13195-025-01820-9. 40783766 PMC12335041

[85] Liu W, Yu L, Chen Q, Zhang C, Wang L, Yu N, Peng D, Ou J, Chen W, Zhang Y, et al. *Poria cocos* polysaccharides alleviate obesity-related adipose tissue insulin resistance via gut microbiota-derived short-chain fatty acids activation of FGF21/PI3K/AKT signaling. Food Res Int. 2025;215(1):116671. doi: 10.1016/j.foodres.2025.116671. 40484558

[86] Astbury S, Atallah E, Vijay A, Aithal GP, Grove JI, Valdes AM. Lower gut microbiome diversity and higher abundance of proinflammatory genus *Collinsella* are associated with biopsy-proven nonalcoholic steatohepatitis. Gut Microbes. 2020;11(3):569–580. doi: 10.1080/19490976.2019.1681861. 31696774 PMC7524262

[87] An S, Gong X, Zhao L, Jian J, Guo Y, Yang X, Sun H, Li Y, Liu B. Significant changes in gut microbiota and SCFAs among patients with newly diagnosed acute myeloid leukemia. Front Microbiol. 2025;16(1):1559033. doi: 10.3389/fmicb.2025.1559033. 40236478 PMC11997447

[88] Patel BD, Kulkarni G, Chowdhuri S, Arya A, John KM, Doshi ASP, Nair R, Korukonda K. Evaluation of *Streptococcus faecalis*, *Clostridium butyricum*, *Bacillus mesentericus*, *Lactobacillus sporogenes*, *Saccharomyces Boulardi* multistrain probiotic formulation in acute gastroenteritis: a real-world observational study (MAESTRO). BMC Nutr. 2025;11(1):145. doi: 10.1186/s40795-025-01127-w. 40713758 PMC12291354

[89] Périchon B, Cokelaer T, Teh WK, du Merle L, Ma L, Touchon M, Doloy A, Poyart C, Givskov M, Trieu-Cuot P, et al. Colorectal cancer-associated Streptococcus gallolyticus: a hidden diversity expose. J Bacteriol. 2025;207(9):e0023025. doi: 10.1128/jb.00230-25. 40810579 PMC12445087

[90] Liu S, Liu X, Guo J, Yang R, Wang H, Sun Y, Chen B, Dong R. The association between microplastics and microbiota in placentas and meconium: the first evidence in humans. Environ Sci Technol. 2023;57(46):17774–17785. doi: 10.1021/acs.est.2c04706. 36269573

[91] Del Chierico F, Nobili V, Vernocchi P, Russo A, De Stefanis C, Gnani D, Furlanello C, Zandonà A, Paci P, Capuani G, et al. Gut microbiota profiling of pediatric nonalcoholic fatty liver disease and obese patients unveiled by an integrated meta-omics-based approach. Hepatology. 2017;65(2):451–464. doi: 10.1002/hep.28572. 27028797

[92] Yang J, Wei H, Zhou Y, Szeto CH, Li C, Lin Y, Coker OO, Lau HCH, Chan AWH, Sung JJY, et al. High-fat diet promotes colorectal tumorigenesis through modulating gut microbiota and metabolites. Gastroenterology. 2022;162(1):135–149. doi: 10.1053/j.gastro.2021.08.041. 34461052

[93] Chanda D, De D. Meta-analysis reveals obesity associated gut microbial alteration patterns and reproducible contributors of functional shift. Gut Microbes. 2024;16(1):2304900. doi: 10.1080/19490976.2024.2304900. 38265338 PMC10810176

[94] Parker BJ, Wearsch PA, Veloo ACM, Rodriguez-Palacios A. The genus *Alistipes*: gut bacteria with emerging implications to inflammation, cancer, and mental health. Front Immunol. 2020;11(1):906. doi: 10.3389/fimmu.2020.00906. 32582143 PMC7296073

[95] Peng Y, Lu J, Fan L, Dong W, Jiang M. Simulated gastrointestinal digestion of two different sources of biodegradable microplastics and the influence on gut microbiota. Food Chem Toxicol. 2024;185:114474. doi: 10.1016/j.fct.2024.114474. 38301992

[96] De Filippis F, Pasolli E, Ercolini D. Newly explored *Faecalibacterium* diversity is connected to age, lifestyle, geography, and disease. Curr Biol. 2020;30(24):4932–4943. doi: 10.1016/j.cub.2020.09.063. 33065016

[97] Sokol H, Pigneur B, Watterlot L, Lakhdari O, Bermúdez-Humarán LG, Gratadoux JJ, Blugeon S, Bridonneau C, Furet JP, Corthier G, et al. *Faecalibacterium prausnitzii* is an anti-inflammatory commensal bacterium identified by gut microbiota analysis of Crohn disease patients. Proc Natl Acad Sci U S A. 2008;105(43):16731–16736. doi: 10.1073/pnas.0804812105. 18936492 PMC2575488

[98] Assa A, Butcher J, Li J, Elkadri A, Sherman PM, Muise AM, Stintzi A, Mack D. Mucosa-associated ileal microbiota in new-onset pediatric Crohn's disease. Inflamm Bowel Dis. 2016;22(7):1533–1539. doi: 10.1097/MIB.0000000000000776. 27271491

[99] Hansen R, Russell RK, Reiff C, Louis P, McIntosh F, Berry SH, Mukhopadhya I, Bisset WM, Barclay AR, Bishop J, et al. Microbiota of de-novo pediatric IBD: increased *Faecalibacterium prausnitzii* and reduced bacterial diversity in Crohn's but not in ulcerative colitis. Am J Gastroenterol. 2012;107(12):1913–1922. doi: 10.1038/ajg.2012.335. 23044767

[100] Durazzi F, Sala C, Castellani G, Manfreda G, Remondini D, De Cesare A. Comparison between 16S rRNA and shotgun sequencing data for the taxonomic characterization of the gut microbiota. Sci Rep. 2021;11(1):3030. doi: 10.1038/s41598-021-82726-y. 33542369 PMC7862389

[101] Zha H, Lv J, Lou Y, Wo W, Xia J, Li S, Zhuge A, Tang R, Si N, Hu Z, et al. Alterations of gut and oral microbiota in the individuals consuming take-away food in disposable plastic containers. J Hazard Mater. 2023;441(1):129903. doi: 10.1016/j.jhazmat.2022.129903. 36087528

[102] Zhao J, Zhang H, Shi L, Jia Y, Sheng H. Detection and quantification of microplastics in various types of human tumor tissues. Ecotoxicol Environ Saf. 2024;283(1):116818. doi: 10.1016/j.ecoenv.2024.116818. 39083862

[103] Ibrahim YS, Tuan Anuar S, Azmi AA, Wan Mohd Khalik WMA, Lehata S, Hamzah SR, Ismail D, Ma ZF, Dzulkarnaen A, Zakaria Z, et al. Detection of microplastics in human colectomy specimens. JGH Open. 2020;5(1):116–121. doi: 10.1002/jgh3.12457. 33490620 PMC7812470

[104] Pan W, Hao J, Zhang M, Liu H, Tian F, Zhang X, Jiang Z, Chen C, Gao M, Zhang H. Identification and analysis of microplastics in peritumoral and tumor tissues of colorectal cancer. Sci Rep. 2025;15(1):16130. doi: 10.1038/s41598-025-98268-6. 40341187 PMC12062370

[105] Hu D, Liu H, Guo Y, Zhang H, Qiu M, Yang Z, Hao J, Jiang Z, Gao M, Zhang X, et al. Microplastics promote chemoresistance by mediating lipid metabolism and suppressing pyroptosis in colorectal cancer. Apoptosis. 2025;30:2287–2300. doi: 10.1007/s10495-025-02143-8. 40681800

[106] Yan Z, Liu Y, Zhang T, Zhang F, Ren H, Zhang Y. Analysis of microplastics in human feces reveals a correlation between fecal microplastics and inflammatory bowel disease status. Environ Sci Technol. 2022;56(1):414–421. doi: 10.1021/acs.est.1c03924. 34935363

[107] Wu F, Wu F, Liu X, Xie W, Liang Y, Ye Y, Xiao X, Sun K, Bai L, Liu S, et al. Microplastic accumulation in fibrotic intestinal tissue and mesenteric adipose tissue in Crohn's disease patients. Environ Res. 2025;271:121077. doi: 10.1016/j.envres.2025.121077. 39947377

[108] Kuroiwa M, Yamaguchi SI, Kato Y, Hori A, Toyoura S, Nakahara M, Morimoto N, Nakayama M. Tim4, a macrophage receptor for apoptotic cells, binds polystyrene microplastics via aromatic-aromatic interactions. Sci Total Environ. 2023;875(1):162586. doi: 10.1016/j.scitotenv.2023.162586. 36871719

[109] Ghosal S, Bag S, Rao SR, Bhowmik S. Exposure to polyethylene microplastics exacerbate inflammatory bowel disease tightly associated with intestinal gut microflora. RSC Adv. 2024;14(35):25130–25148. doi: 10.1039/d4ra04544k. 39139248 PMC11320195

[110] Zolotova N, Silina M, Dzhalilova D, Tsvetkov I, Fokichev N, Makarova O. Influence of microplastics on manifestations of experimental chronic Colitis. Toxics. 2025;13(8):701. doi: 10.3390/toxics13080701. 40863977 PMC12390349

[111] Liu Z, Sokratian A, Duda AM, Xu E, Stanhope C, Fu A, Strader S, Li H, Yuan Y, Bobay BG, et al. Anionic nanoplastic contaminants promote Parkinson's disease-associated α-synuclein aggregation. Sci Adv. 2023;9(46):eadi8716. doi: 10.1126/sciadv.adi8716. 37976362 PMC10656074

[112] Xia S, Yan C, Cai G, Xu Q, Zou H, Gu J, Yuan Y, Liu Z, Bian J. Gut dysbiosis exacerbates inflammatory liver injury induced by environmentally relevant concentrations of nanoplastics via the gut-liver axis. J Environ Sci. 2025;155(1):250–266. doi: 10.1016/j.jes.2024.11.022. 40246463

[113] Cui H, Jiang X, Cao J, Yang W, Yang B, Li M. Comparative analysis of metabolic dysfunctions associated with pristine and aged polyethylene microplastic exposure via the liver-gut axis in mice. ACS Nano. 2025;19(14):14272–14283. doi: 10.1021/acsnano.5c00945. 40189833

[114] Wang X, Deng K, Zhang P, Chen Q, Magnuson JT, Qiu W, Zhou Y. Microplastic-mediated new mechanism of liver damage: from the perspective of the gut-liver axis. Sci Total Environ. 2024 Apr 1;919:170962. doi: 10.1016/j.scitotenv.2024.170962. 38360312

[115] Zhang X, Wang H, Peng S, Kang J, Xie Z, Tang R, Xing Y, He Y, Yuan H, Xie C, et al. Effect of microplastics on nasal and intestinal microbiota of the high-exposure population. Front Public Health. 2022;10(1):1005535. doi: 10.3389/fpubh.2022.1005535. 36388272 PMC9650105

[116] Imhann F, Bonder MJ, Vich Vila A, Fu J, Mujagic Z, Vork L, Tigchelaar EF, Jankipersadsing SA, Cenit MC, Harmsen HJ, et al. Proton pump inhibitors affect the gut microbiome. Gut. 2016;65(5):740–748. doi: 10.1136/gutjnl-2015-310376. 26657899 PMC4853569

[117] Weersma RK, Zhernakova A, Fu J. Interaction between drugs and the gut microbiome. Gut. 2020;69(8):1510–1519. doi: 10.1136/gutjnl-2019-320204. 32409589 PMC7398478

